# Surveying the familiarity and usage of up-to-date database among nurses working in the hospitals affiliated with Ahvaz Jundishapur University of Medical Sciences

**Published:** 2015

**Authors:** AH Ghassemi, E AghazadeAsl, Z Bigdeli, A SakiMalehi

**Affiliations:** * Ahvaz Jundishapur University of Medical Sciences, Iran

**Keywords:** nurses, degree of familiarity, usage, Up-To-Date database, Ahvaz Jundishapur University of Medical Sciences

## Abstract

**Background and Objectives:** The evidence-based databases provide the details of the latest scientific findings of research on an individual subject. Factual databases have gained a significant attention regarding the importance and application of evidence-based medical information. Therefore, the purpose of present study was to investigate the level of familiarity by using an Up-To-Date database among the nurses working at the hospitals affiliated with Ahvaz Jundishapur University of Medial Sciences, and by this, making ground for cost-efficiency and cost-effectiveness analyses.

**Materials and Methods:** This was a descriptive-analytical research, conducted on a sample of 293 nurses selected from a population of 1246 nurses, working at the hospitals affiliated with Ahvaz Jundishapur University of Medical Sciences. For the data collection, a researcher built questionnaire was used, and its validity and reliability were confirmed, its Cronbach’s alpha coefficient being calculated as 0.71. For the data analysis, descriptive statistics and Kruskal-Wallis test were run.

**Results:** According to the findings, the majority of the nurses were female, having a Bachelor’s degree. The nurses had a mid-familiarity with the Up-To-Date database, and most of them used this database in a very limited manner. When using the Up-To-Date database, their primary goal was to update their information and to answer to the clinical questions of the patients.

**Conclusions:** According to the findings, it could be concluded that the access to the recent and up-to-date information in the domain of nursing required the training of the nurses with the necessary skills in information use, to be able to obtain updated information from evidence-based databases and other information resources.

## Background

Nurses require an extensive range of information to meet their educational and clinical needs, as well as to improve the quality of the medical services. It is clear that due to different reasons, nurses cannot rely only on their professional experiences, and may obtain this type of information from trustable ways. As a result, they use the experiences of their colleagues, doctors, the others in the medical team, the library resources (printed or online), and the field-related databases. 

Online databases provide access to a large amount of qualitative and quantitative information in a rapid, precise, and integrated way [**1**]. Selecting an appropriate database is necessary to meet the information needs of the consumer community (here, the nursing community). Among the databases that are available in the nursing domain, there is the Up-To-Date database, a medicine and nursing related database, which provides various services in these fields. These include the latest information and clinical findings on diseases, treatment methods, new approaches for caring patients, professional behavior toward patients, and the pharmaceutical information provided by expert physicians. Hence, Up-To-Date database could be considered among the most useful databases in the domain of nursing in the provision of such a service to users. 

Up-To-Date database includes the peer-reviewed information resources and evidence-based medical documents which contain detailed information on caring methods, patients’ clinical issues (clinical symptoms, new laboratory methods, diagnosis, and treatment of disease) in the form of articles, as well as pharmaceutical information and drug interactions. These types of information are useful for physicians, nurses, patients, and other professionals related to the health and care domain. According to some users, this database is the sole resource that can support decision-making processes about the improvement of the inevitable outcomes [**2**-**4**]. 

Since the Up-To-Date database contains information and resources that are derived from evidence-based activities, it tries to provide scientific data for users (physicians, nurses, patients, etc.) through combining the best knowledge available from evidence, clinical experiences, and the patients’ values. 

Research has shown that the nurses who worked based on scientific evidence, however, were able to make better decisions, take higher quality care, reduce the term of hospitalization and the costs for the patient, and provide a better cost-effectiveness for the patient and the organization. These nurses have been able to pay attention to clients (patients) and give them reliable and appropriate answers [**5**].

The Up-To-Date database can have a significant role in the field of nursing, because this database provides nurses with the updated, new, evidence-based, practical, comprehensive, and quick information that are useful in decision-making, providing service to patients and avoiding the wasting of time in caring the patients, and also prevent drug side-effects. It also resulted in an effective communication with the patients, the ability to respond to clinical questions, and deal with clinical issues. It helped the nurses to sort out the best research evidence, and clinical practices combined with the patient’s values and offer the best service to the patient. Since medical texts become obsolete and outdated with the passing of time, the Up-To-Date database can provide new information to new scientific evidence to solve this problem. 

Given that the Up-To-Date database was subscribed by the Ministry of Health and Medical Education, by costing ideal for feeding and purchasing the subscription to this database on one hand, and since this database provides updated information in the fields of medicine and nursing that are necessary for providing high-quality services to the contrary, this database can be used by all universities under the support of the Ministry of Health and Medical Education. However, no research has addressed how much and in what ways nurses (as one of the most important professions involved in health care) use the Up-To-date database; hence, the nurse’s familiarity and use of this database is not clear.

It is noteworthy that the subscription to this database costs significantly to the Ministry of Health and Medical Education (MHME), not mentioning the long and complicated process of subscription. Thus, regarding the cost and facilities spent for this database, it is expected that the medical society maximally used the database; which first requires the awareness of educational and medical centers affiliated to the universities towards this data bank, and clarifying their actual use of the database. Without having enough information on the familiarity with and the real use of this data bank, universities and colleges will not be able to plan properly for the efficient use of the facilities and resources spent in this regard. It is also necessary to note that having access to a variety of information sources and databases, especially the Up-To-Date database, is a requirement in the modern education and health system in the universities affiliated to the MHME. The access to new information and its use to update the information not only promotes health and education users especially nurses but also increases significantly the quality of health care provided for the patients.

## Methods

The existing study was a descriptive-analytical research that examined and analyzed the nurses’ acquaintance and use of the Up-To-Date database in the hospitals affiliated with Jundishapur University of Medical Sciences. The study population included all the nurses working in hospitals affiliated with Jundishapur University of Medical Sciences in Ahvaz city. According to the statistics, the number of employed nurses was 1246 in total, among which, 293 nurses were determined as the sample of the study by using Krejcie and Morgan table. The data collection tool was a researcher built questionnaire, adapted from Rahimianfar, et al. [**15**] and Darzi [**1**]. The data were analyzed by using SPSS. To demonstrate the demographic features of the respondents and also to find the relationship among the research variables, variance analysis and Kruskall-Wallis test was run. 

**Research findings **

Descriptive statistics 

**Table 1:** Demographic features

Variables		Number of respondents (n)	Frequency (%)
Hospital's name	Emam	59	23.3
	Golestan	53	20.7
	Abuzar	46	18.9
	Razi	33	13
	Sina	21	8.3
	Shafa	15	5.9
	Taleqani	15	5.9
	Salamat	10	4
Gender	Female	206	81.4
	Male	46	18.2
Level of education	Associate diploma	7	2.8
	Bachelor’s	233	92.5
	Master’s	12	4.8
Employment status	Temporary	75	29.6
	Contractual	67	26.5
	Project-based	63	24.9
	Permanent	48	19

**Inferential statistics**

1. To what degree did the nurses working in the hospitals affiliated with Jundishapur University of Medical Sciences were familiar with the Up-To-Date database?

**Table 2:** Percentage and frequency of the nurses’ familiarity with the Up-To-Date database

Familiarity	Frequency	Percentage
Good	20	7.9
Average	107	42.3
Little	85	33.6
Very little	41	16.2
Total	253	100

Regarding the first research question and the results presented in the **table** above, it was found that among the responding nurses, none had an excellent familiarity with the Up-To-Date database. 

2. How did the nurses working in the hospitals affiliated with Jundishapur University of Medical Sciences become familiar with the Up-To-Date database?

**Table 3:** Percentage and frequency of the nurses’ way of familiarity with the Up-To-Date database

Way of familiarity with the Up-To-Date database	Number	Percentage
Searching the internet and electronic resources	82	32.4
Brochures and guidelines	78	30.8
Doctors and colleagues	71	28.1
During education at the university	63	24.9
Hospital’s library personnel	49	19.4
Central Library website	30	11.9
Books and magazines	15	5.9
Specialized in-hospital courses	13	5.1
Free courses	3	1.2

To find out how nurses have become familiar with the Up-To-Date database, the results presented in **Table 3** indicate that the familiarity was achieved through searching the web and the electronic sources for 82 nurses (32.4%), reading brochures and guidelines for 78 nurses (30.8%), information from doctors and colleagues for 71 nurses (28.1%), during their academic education 63 nurses (24.9%), have been the most frequent ways of getting familiar with the Up-To-Date database.

3. How frequently did the nurses working in the hospitals affiliated with Jundishapur University of Medical Sciences use the Up-To-Date database for their information needs? 

**Table 4:** Percentage and frequency of the nurses’ use of Up-To-Date database

Use	Frequency	Percentage
Mostly	9	3.6
On average	74	29.2
Little	99	39.1
Very little	71	28.1
Total	253	100

To find out the answer to the third research question, the results presented in **Table 4** show that the Up-To-Date database is mostly used only by 9 nurses (3.6%). 

4. Was there any relationship between the nurses’ academic degree and their use of the Up-To-Date database?

**Table 5:** The relationship between the level of education with the familiarity and use of the Up-To-Date database by nurses

Academic degree	N	Mean	Sd	Min	Max	P
Associate diploma	7	8.1429	2.26	5	12	0.023
Bachelor’s	223	8.5291	2.91	4	16	
Master’s	9	11.4444	3.53	4	16	

To find the relationship between the education level and the familiarity with the Up-To-Date database, Kruskal-Wallis test was used. According to the results presented in **Table 5**, it could be concluded that a significant relationship existed among the familiarity of nurses with the Up-To-Date database and using this database in different educational levels. 

5. What were the most important goals of nurses working in the hospitals affiliated with Jundishapur University of Medical Sciences in using the Up-To-Date database?

**Table 6:** Frequency and percentage of the nurses’ motivation and goal from using the Up-To-Date database

Motivation and goal	N	Percentage
Updating personal information	139	54.9
Responding to clinical questions	105	41.5
Establishing scientific relationships	90	35.6
Awareness of the new diseases	51	20.2
Research and study	50	19.8
Increasing knowledge	35	13.8
Preparing for academic tests	32	12.6
Awareness of new health and care methods and treatments	16	6.3

To answer the fifth question, the results presented in **Table 6** were the main reasons of the respondents in referring to the Up-To-Date database were to update the personal information (54.9%), respond to clinical questions (41.5%), and establish a clear relation with the other colleagues and doctors in the world (35.6%). 

## Discussion 

The nursing profession is moving toward evidence-based care, and nurses, as the largest group in the health profession, are required to use information resources, visit the libraries and information centers more than others. Since literature can have a very positive effect in the treatment of patients, it is expected that nurses make their clinical decisions based on the evidence from new valid studies. 

The Up-To-Date database is one of the most comprehensive and updated databases that provides evidence-based information needed for the care of patients, thus playing a significant role in the nursing profession and helping them to provide better services suited to the caring needs of patients, and also the information needs of nurses. 

Our findings on the degree of familiarity of the nurses with the Up-To-Date database in the hospitals affiliated with the Medical University of Ahvaz presented in **Table 2**, showed that about 49.9% of them had little and close to no familiarity at all with this data bank. According to **Table 4**, most of them (67.2%) used this database at a low level (39.1%). Since no independent research was performed in this domain and specifically in this data bank, the findings of this study were compared with the other populations and subjects. The results of this examination were congruent with some studies as it follows: Kosteniuk [**12**] pointed the small level of familiarity and use of electronic sources and specialized medical information banks by nurses compared with library documents, Habibi et al. [**17**] found that one-fourth of the respondents used information databases especially Medline, and others did not use databanks and databases, even electronic journals, very much, Rahimianfar et al. [**14**] pointed a 18.7% use of the information banks by the nurses, Griffiths and Riddington [**18**] reported a lack of awareness among the majority of the nurses on the electronic resources and information banks in their specialized fields.

The Up-To-Date database is an evidence-based information center that includes new information and findings, and our results indicated that nurses were not aware of the facilities and information provided by this database and were ignorant of the advantages of such information, which could be useful for the clinical and educational work of nurses. Its use was slight regarding the money spent for providing access to this information source. However, this amount of expense requires more attention and maximum use by the nurses. 

Furthermore, findings represented in **Table 5** showed that the familiarity and use of the Up-To-Date database had a significant relationship with the academic degree. This was consistent with Mohajeri and Alijanpour & Kasgari [**10**] study in which they underlined the level of familiarity and use of online information databases by the students of Babul Medical University. It was also consistent with the studies of Shakouri Monfared [**6**] and Rahimianfar et al. [**15**].

The findings related to the familiarity of nurses with the Up-To-Date database provided in **Table 3**, indicated that most of the nurses became familiar with this database through electronic resources (32.4%), reading the brochures and guidelines (30.8%), and through the help of doctors and colleagues (28.1%). This was consistent with the findings of Darzi [**1**]. The results of the research also showed that the familiarity with the database as a result of the hospitals’ and libraries’ instructions was little. This table also showed that most of the nurses knew about the Up-To-Date database thanks to the Internet and electronic resources.

Results presented in **Table 6** showed the motivation and objective of nurses in using the Up-To-Date database. It indicated that most of the nurses used this database with the aim of updating their knowledge and being able to answer the clinical questions of patients. This finding was consistent with Tomas’s [**19**] who mentioned finding drug-related information and policies of the nursing profession as the main reasons for seeking information in the clinical environments. This finding was also mentioned by Rahimianfar et al. [**15**]. Hence, in the present study, it was mentioned that the objective and motivation of nurses was essential for the efficient use of the database; because if people had a particular purpose and motivation for seeking information in the databases and other resources, they would use the resource more frequently. In other words, paying attention to the motives and goals of users in referring to an information source and sharing it increases the utility of the source and also gives economic justification and cost-effectiveness to the process. Moreover, the goals of people affect the use of information resources because it might happen that an individual looks for a certain kind of information but cannot find it in the intended database and after that, he would stop using that data bank. Hence, a constant review of the users’ objectives in using an information source promotes and extends the applications of that source, and enhances the common grounds in the users’ goals, and the capacity of that database. 

## Conclusion

One way of achieving scientific and technical information is by using online databases. The databases that are available online through universities are prestigious and influential sources of information for research and the information needs of professionals. The practical use of these sites depends on the familiarity with them and having information-seeking skills, which can be acquired through training courses on the use of databases. Also, evidence-based information resources present the latest research findings in the least possible time to the users and give them the opportunity to update their information and meet their information needs based on the updated sources and take preventive actions in the face of possible problems. 

The nature of nursing profession requires its practitioners to have access to up-to-date and useful sources to make appropriate decisions. Also, they should use this information for the evaluating of the information of patients in critical situations, prescription of drugs, finding official rules and regulations, and discovering the related progresses in the health care domain. Evidence-based information helps the nurses to update their knowledge based on the latest changes and findings and accordingly improve their services including prevention, treatment, diagnosis, and care.

Since many information centers present up to date information in the medical and related fields, the healthcare professionals became confused in finding information in these databases. The fact that information quickly becomes outdated and even rejected in the medical sciences may inevitably cause the society of medical professionals to have problems in finding the latest information and use of databases. Hence, as an evidence-based information database, the Up-To-Date database has many applications in different domains of medical sciences and provides the newest information in this regard. It prevents the waste of the time and energy of the medical society and nurses significantly, so that the nurses could obtain the necessary information to have a good communication with their patients and provide care services to them. It also gives the opportunity to the patients to become aware of the type of their disease, and advantages and disadvantages of each treatment procedure and helps nurses and doctors in the process of therapy. 

The present study investigated the level of familiarity with the use of the Up-To-Date database by nurses, their motivations and objectives in using this database, and how they became familiar with the Up-To-Date database. The results of this study showed that nurses did not use this database frequently, but also had little knowledge about the facilities, information, and services provided by it. The reason for this issue was the lack of a proper introduction of the database, lack of related education, and not being informed regarding the applications of this database. 

**Suggestions**

Regarding the results of this study it was suggested that:

- According to **Table 5**, since there was a significant relationship between the level of education and familiarity of the nurses in using the Up-To-Date database, it was suggested that workshops and introductory courses should be held for nurses at various educational levels. If these seminars continued for a period, nurses would be more motivated to attend them. 

- According to **Table 3**, since the majority of nurses became familiar with the Up-To-Date database by surfing the web and electronic resources and less through courses held by the central library and the hospital’s library or the academic terms in the university, it was suggested that schools added some classes for the introduction of information databases related to the medical fields. 

- Since brochures and advertising posters have a significant role in the people’s awareness, **Table 3** in this study also showing that brochures have a greater role in the nurses’ familiarity with the Up-To-Date database, it was suggested that leaflets and brochures were prepared by the authorities and were distributed in the hospitals and health centers as well as at the university level, stating the advantages of using this database, the information contained in the database, and also how to search and find information in this database. 

- The level of use and familiarity of the academicians and professors of nursing also had a major role in making nurses familiar with the database. Here, as the university professors were more capable in the education, students could better learn how to work with this database and use it. Therefore, it was suggested that courses had to be held for academicians and practitioners and other professionals involved in training nurses so that nurses were better acquainted with the Up-To-Date database.

- Research on the level of familiarity and use of the Up-To-Date database by the university professors, doctors, and related personnel such as hospital mentors, officials, librarians, and information centers could be conducted. Because a good understanding of the extent to which university professors and doctors used this database helped in identifying their information needs and potential problems compared to those of nurses.

**Acknowledgements**

The authors would like to thank Ahvaz nurses working in hospitals affiliated to Jundishapur University of Medical Sciences for their participation in this study and their help in writing this article.

## References

[1] Darzi S (2011). Mazandaran University graduate students’ information seeking behavior in the use of online information resources. Quarterly of Information Science (Library and Information Science and Technology).

[2] (2015). The up-to-date database, Shahed University, Central Library and Information Center.

[3] Up-to-date database, access form Gatepaper.ir.

[4] Up-to-date database, Central Library of Ahvaz University of Medical Sciences, the guideline to electronic resources.

[5] Adib Haj Baqeri M (2006). The effective factors on evidence-based nursing care: a qualitative study. Quarterly of Nursing in Iran.

[6] Shakouri Monfared H (1993). Investigating the information needs of nurses working in Iran medical universities of Iran, Tehran, and Shahid Beheshti (thesis).

[7] Torabi L (2001). Evaluation of the use of electronic medical sources in central libraries and departments of public medical universities in Tehran from the viewpoint of librarians (thesis).

[8] Asemi A, Riyahinia N (2007). Awareness and use of digital resources in the libraries of Isfahan University of Medical Sciences, Iran.

[9] Hashemian MR, Janatikia  M, Hashemian A (2013). Information seeking skills in online digital databases available at the National Library of Medicine among Medical Assistants in the Medical University of Isfahan. Health Information Management.

[10] Mohajeri F, Alijanpoor Kasgari  M (2010). Evaluating the familiarity with and use of the online database by students of Babul Medical University. Journal of Information Knowledge.

[11] Tannery NH, Wessel ChB, Epstein BA, Gadd CS (2007). Hospital nurse’s use of knowledge-based information resources. Nursing Outlook.

[12] Kosteniuk  JG, D’Arcy C, Stewart  N, Smith  B (2006). Central and peripheral information source use among rural and remote registered nurses. Journal of Advanced Nursing.

[13] Dee  C, Stanley  EE (2005 ). Information-seeking behavior of nursing students and clinical nurses: implications for health sciences librarians. Journal of Medical Library Association.

[14] Randell  D, Mitchell  N, Thompson  C, Mc Caughan  D, Dowding  D (2009 ). From pull to push: understanding nurses’ information needs. Health Informatics Journal.

[15] Rahimianfar A, Hakimian R, Salimi T (2013). Evaluating the information seeking behavior of nurses working in educational hospitals of Yazd city in 2011. Health Information Management.

[16] Nahrir  B, Rejeh  N, Ebadi  A (2013). Teaching evidence-based nursing. Nursing Education.

[17] Habibi Sh, Farzi  J, Lotfollahzadeh R (2008). Information seeking behavior of physicians in Ardebil city and their approach to electronic sources. Journal of Ardebil University of Medical Sciences.

[18] Griffiths P, Riddington L (2001). Nurses’ use of computer databases to identify evidence for practice—a cross-sectional questionnaire survey in a UK hospital. Health Information and Libraries Journal.

[19] Thomas S (2012). A case study of the information needs of nurses at a university hospital trust in the East Midlands: looking towards how information provision could improve (Thesis).

